# Transferrin is responsible for mediating the effects of iron ions on the regulation of anterior pharynx-defective-1α/β and Presenilin 1 expression via PGE_2_ and PGD_2_ at the early stage of Alzheimer’s Disease

**DOI:** 10.18632/aging.101615

**Published:** 2018-11-01

**Authors:** Chen-Di Lu, Ji-Kang Ma, Zheng-Yang Luo, Qun-Xi Tai, Pu Wang, Pei-Pei Guan

**Affiliations:** 1 College of Life and Health Sciences, Northeastern University, Shenyang 110819, P. R. China

**Keywords:** transferrin, transferrin receptors, iron ions, cyclooxygenase-2, peroxisome proliferator-activated receptor γ

## Abstract

Transferrin (Tf) is an important iron-binding protein postulated to play a key role in iron ion (Fe) absorption via the Tf receptor (TfR), which potentially contributes to the pathogenesis of Alzheimer’s disease (AD). However, the role of Tf in AD remains unknown. Using mouse-derived neurons and APP/PS1 transgenic (Tg) mice as model systems, we firstly revealed the mechanisms of APH-1α/1β and presenilin 1 (PS1) upregulation by Fe in prostaglandin (PG) E_2_- and PGD_2_-dependent mechanisms. Specifically, Fe stimulated the expression of mPGES-1 and the production of PGE_2_ and PGD_2_ via the Tf and TfR system. Highly accumulated PGE_2_ markedly induced the expression of anterior pharynx-defective-1α and -1β (APH-1α/1β) and PS1 via an EP receptor-dependent mechanism. In contrast, PGD_2_ suppressed the expression of APH-1α/1β and PS1 via a prostaglandin D_2_ (DP) receptor-dependent mechanism. As the natural dehydrated product of PGD_2_, 15d-PGJ_2_ exerts inhibitory effects on the expression of APH-1α/1β and PS1 in a peroxisome proliferator-activated receptor (PPAR) γ-dependent manner. The expression of APH-1α/1β and PS1 ultimately determined the production and deposition of β-amyloid protein (Aβ), an effect that potentially contributes to the pathogenesis of AD.

## Introduction

Iron (Fe) ions are metal ions that are widely investigated in the pathogenesis of Alzheimer’s disease (AD). Elevated Fe in AD brains was firstly identified in 1953 and has been shown to be associated with β-amyloid plaques (APs) [1] and neurofibrillary tangles (NFTs), which indicate that Fe chelator therapy may be a new target for AD [2]. Fe load was also reported to be elevated in the frontal cortex of 12-month-old APP/PS1 Tg mice [3]. In SH-SY5Y cells, it is reported that overloading of Fe is caused by the production of β -amyloid protein (Aβ) [4]. In addition, Fe can bind to Aβ [5,6] and to tau [7]. The binding of iron to Aβ or to tau induces the aggregation of Aβ [8] and the hyperphosphorylation of tau [9] to form APs and NFTs. The binding of iron to Aβ or to tau also enhances the toxicity of Aβ [10,11] and tau to neurons [2,12]. Indeed, iron-aggregated Aβ is mediated by Reactive oxygen species (ROS) [13] or by the activation of the Bcl-2-related apoptotic pathway [14]. In addition, Egana *et al*. [15] reported that the treatment of cultured hippocampal neurons with Fe decreases tau phosphorylation. In contrast, Fe treatment has been shown to increase the phosphorylation of tau [16,17], which may be caused by the upstream activation of the ERK1/2 pathway [18,19].

In light of the critical roles of Fe in AD, Fe transporters have received a great deal of attention. For example, transferrin (Tf) constitutes a family of Fe-binding proteins that transport Fe into the endosomal compartment of cells by forming complexes of Fe-bound Tf and the Tf receptor (TfR) [20]. Upon maturation, the endosome is acidified, and Fe is released from transferrin and then transported to the cytosol by a divalent metal transporter 1 (DMT1) [21]. Once Fe is released from transferrin, it is known to induce the generation of oxygen radicals, which in turn stimulate the production of toxic Aβ oligomers [22]. Reciprocally, the toxicity of Fe is elevated when Aβ oligomers are stabilized [10]. In addition, Fe accumulation delays the formation of allegedly less toxic, well-ordered aggregates [10]. In contrast to these observations [10,22], Fe sequestration by Tf reduces Aβ self-association [23,24]. On the one hand, Tf sequesters Fe in a relatively nonreactive and inert state and inhibits Aβ aggregation. On the other hand, the transportation and release of Fe by Tf will facilitate the stabilization and toxicity of Aβ oligomers.

Although the role of Tf in the pathogenesis of AD remain thoroughly clear, Fe may potentially contribute to the expression and metabolic activity of COX-2 in rats with diabetic nephropathy [22]. This observation provides clues for deciphering the mechanism of Fe in Aβ production and deposition. As expected, COX-2 is involved in modulating the production and aggregation of Aβ during the course of AD development. For example, Gasparini *et al.* [25] reported that exogenous treatment of neuroblastoma 2a (n2a) cells and rat primary cortical neurons with inhibitors of COX-2, including flurbiprofen and sulindac sulfide, decreased the secretion of Aβ_1-42_ and Aβ_1-40_ in a dose-dependent manner. This *in vitro* observation was further confirmed in APP/PS1 Tg mice [26]. To exclude the nonspecificity of COX-2 inhibitors, Xiang *et al.* [27] revealed that human COX-2 expression in APP/PS1/COX-2 mice induced potentiation of brain parenchymal amyloid plaque (AP) formation and produced a greater than 2-fold increase in PGE_2_ production at the age of 24 months. In line with these *in vitro* observations [27], Akitake *et al.* [28] suggested that mPGES-1, a PGE_2_ synthase, is induced in human AD patients and the Tg2576 mouse, a transgenic AD mouse model. Knockout of the mPGES-1 gene reduces the accumulation of microglia around APs and attenuates learning impairments in Tg2576 mice. All of these investigations focused on the important roles of COX-2 and PGE_2_ in aggravating AD.

Apart from COX-2 and its metabolic products, questions have arisen regarding whether Fe elevation regulates the expression of α-, β-, or γ-secretase, the expression of which is involved in Aβ deposition and AD progression. To this end, Guo *et al.* [29] reported that treatment of APP/PS1 Tg mice for 3 months with Fe concurrently increased the expression of β -secretase 1 (BACE-1) and Presenilin 1 (PS1) and decreased the expression of a disintegrin and metallopeptidase domain 10 (ADAM-10), resulting in the production of Aβ during the course of AD development and progression. In contrast, Fe treatment does not show stimulatory effects on the expression of BACE-1 [30] or inhibitory effects on the expression of ADAM-10 in PC12 cells [31]. Based on these observations, it is necessary to identify or confirm the secretases that are regulated by the accumulation of Fe during the course of AD development and progression.

Although Fe has shown its effects on regulating the expression of ADAM-10, BACE-1 and PS1, its roles in the activity of anterior pharynx-defective-1 (APH-1) has been largely overlooked. APH-1 was already reported to combine with PEN-2, nicastrin (NCT), and PS, to generate an active form of the γ-secretase complex that cleaves β-APP and deposits Aβ (8). In addition, APH-1α and -1β are necessary for notch pathway signaling, γ-secretase cleavage of β -amyloid precursor protein (β-APP), and Aβ protein accumulation in C. elegans (7). In line with these studies, our data demonstrated that APH-1α/1β and PS1 were upregulated by Fe via a Tf-dependent mechanisms. In addition, Tf mediated the effects of Fe on stimulating the expression and metabolic activity of mPGES-1 and the production of PGE_2_ and PGD_2_, the production of which antagonistically regulates the expression of APH-1α/1β and PS1 in an EP2- and prostaglandin D_2_ receptor 1 (DP1)-dependent manner. As the natural dehydrated product of PGD_2_, 15d-PGJ_2_ exerts inhibitory effects on the expression of APH-1α/1β and PS1 in a peroxisome proliferator-activated receptor (PPAR) γ-dependent manner, the expression of which potentially contributes to the pathogenesis of AD.

## RESULTS

### Tf and TfR are elevated in the brains of 3-month-old APP/PS1 Tg mice

**Figure 1:**
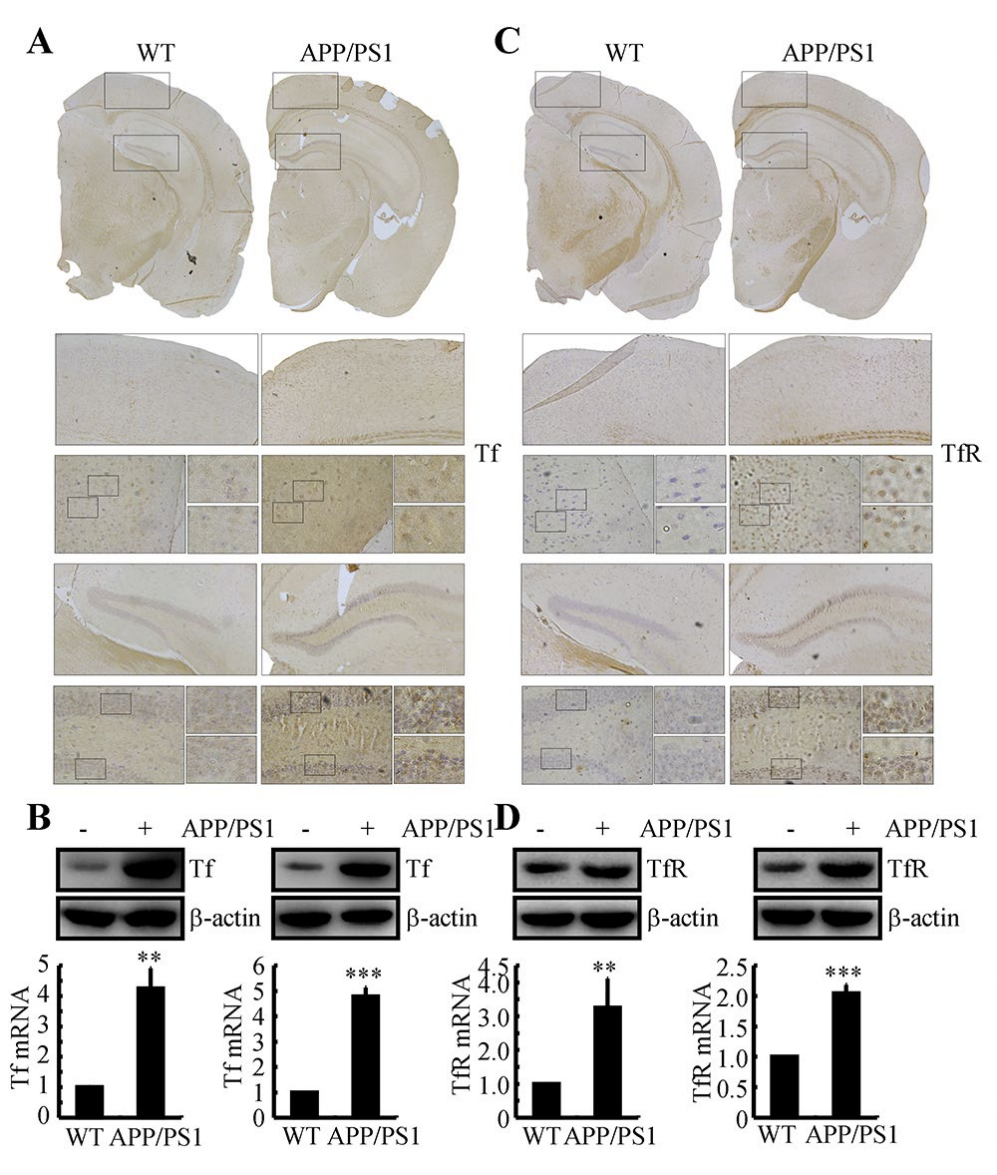
**The expression of Tf and TfR was elevated in 3-month-old APP/PS1 Tg mice.** The brains of 3-month-old APP/PS1 Tg mice were collected after anesthesia and were perfused (n=6). (**A, C**) The immunoreactivity of Tf and TfR was determined by immunohistochemistry with an anti-Tf and anti-TfR antibody, respectively. These images are representative of 6 independent mouse experiments, all of which produced similar results. (**B, D**) The protein expression of Tf and TfR was determined by western blot analysis. β-actin served as the internal control. Tf and TfR mRNA levels were determined by qRT-PCR, with the total amount of GAPDH serving as the internal control. The data represent the means±S.E. of all the experiments. **p<0.01; ***p<0.001 compared with WT controls.

In light of the roles of the Tf and TfR systems in regulating the accumulation of Fe, we were prompted to determine if Tf and TfR are regulated in the brains of APP/PS1 Tg mice. To this end, experiments were carried out to immunostain Tf and TfR in brain slices of 3-month-old APP/PS1 Tg mice. The results demonstrated that Tf and TfR expression levels were elevated in the cerebral cortex and hippocampus of APP/PS1 Tg mice (Figs. 1A, C). To further validate these observations, real-time PCR and western blots experiments were carried out to determine the mRNA and protein expression of Tf and TfR in the cerebral cortex and hippocampus of mice. Similarly, the results demonstrated that the mRNA and protein expression levels of Tf and TfR were increased in 3-month-old APP/PS1 Tg mice compared to those of WT mice (Figs. 1B, D). These observations indicated the possibility that Tf transports Fe into neurons via TfR at the early stage of AD.

### Aβ is responsible for upregulating the expression of APH-1α/1β and PS1 in n2a cells and APP/PS1 Tg mice

**Figure 2:**
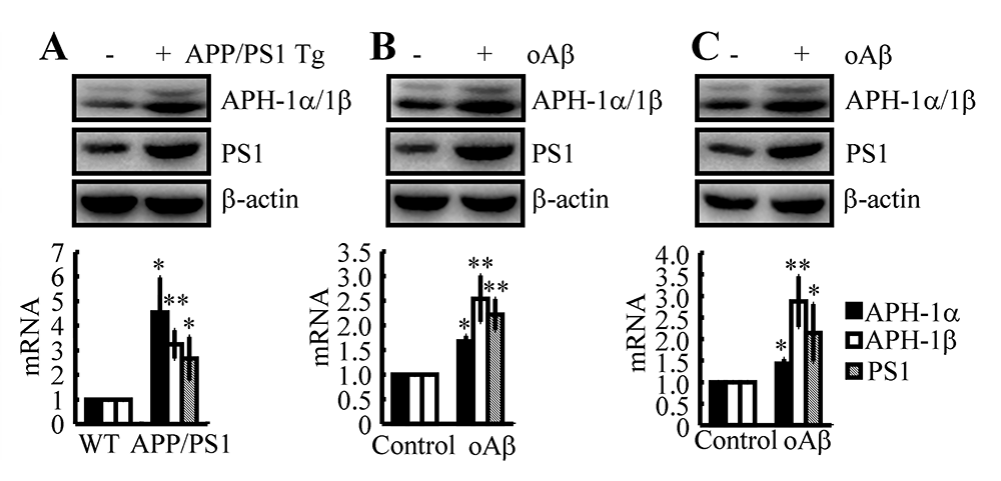
**Aβ plays a critical role in upregulating the expression of APH-1α/1β and PS1 in n2a cells and APP/PS1 Tg mice.** (**A**) The brains of 3-month-old APP/PS1 Tg mice were collected after anesthesia and were perfused (n=12). (**B**) In select experiments, Aβ oligomers (500 ng/5 μl) or vehicle (PBS) was injected (i.c.v) into the ventricles of 3-month-old APP/PS1 Tg mice (n=10). (**C**) In separate experiments, n2a cells were treated with Aβ oligomers (1 μM) for 48 h. (**A-C**) The mRNA and protein levels of APH-1α/1β and PS1 were determined by qRT-PCR and western blots, respectively. The data represent the means±S.E. of all the experiments. *p<0.05; **p<0.01 compared with WT or vehicle-treated controls.

In light of previous studies suggesting that APH-1α/1β and PS1 are involved in the pathogenesis of AD [32,33], we firstly determined the expression levels of APH-1α/1β and PS1 in APP/PS1 Tg mice at 3 months of age. As shown in Fig. 2A, APH-1α/1β and PS1 were markedly upregulated in 3-month-old APP/PS1 Tg mice. To further reveal the cause of the upregulation of APH-1α/1β and PS1, we injected Aβ oligomers (i.c.v, 500 ng/5 μl) into 3-month-old WT mice. The results demonstrated that Aβ oligomers injections (i.c.v, 500 ng/5 μl) significantly stimulated the expression of APH-1α/1β and PS1 in 3-month-old WT mice (Fig. 2B). To further verify the key role of Aβ in upregulating the expression of APH-1α/1β and PS1 *in vivo*, similar experiments were carried out in n2a cells. The results demonstrated that the expression levels of APH-1α/1β and PS1 were elevated in cells stimulated by Aβ oligomers (1 μM) (Fig. 2C). These observations clearly demonstrated that Aβ aggregation is the cause of the synthesis of APH-1α/1β and PS1 during the course of AD development and progression.

### Fe upregulates the expression of APH-1α/1β and PS1 via a COX-2-dependent mechanism in the early stage of AD

**Figure 3:**
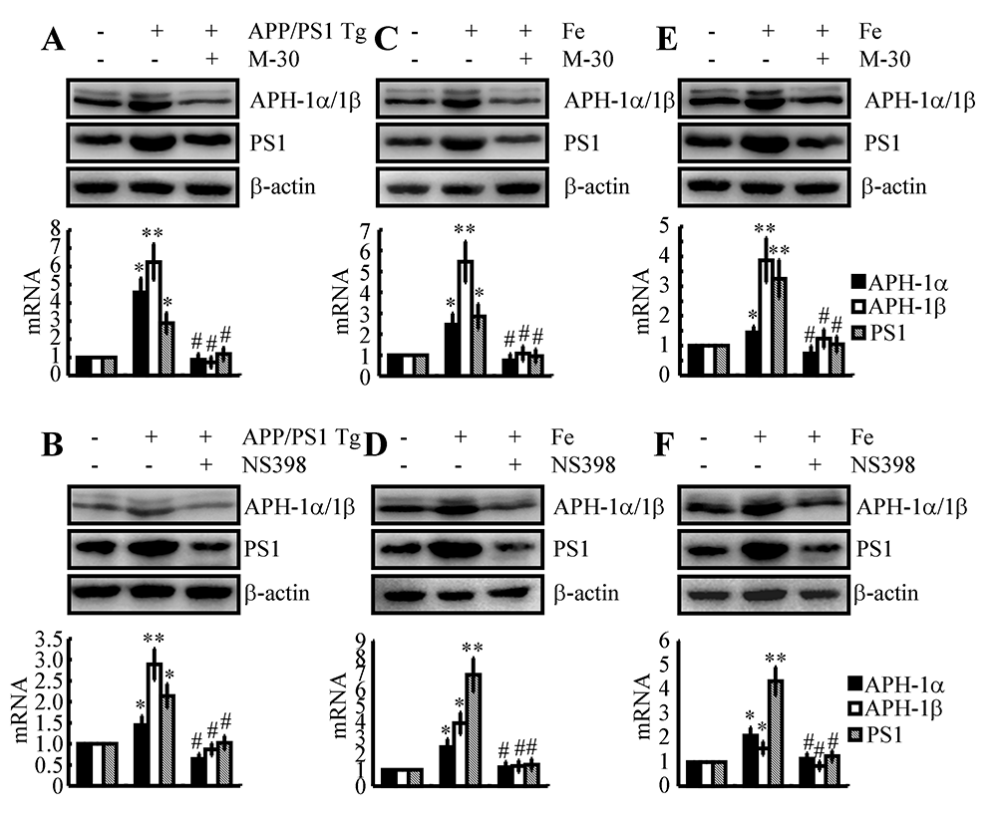
**Involvement of COX-2 in mediating Fe-induced expression of APH-1α/1β and PS1 in neurons.** (**A, B**) APP/PS1 Tg mice were injected (i.c.v) with M-30 (1 μg/5 μl) or NS398 (1 μg/5 μl) for 48 h. (**C, D**) In select experiments, C57BL/6 mice were injected (i.c.v) with Fe (1 μg/5 μl) in the absence or presence of M-30 (1 μg/5 μl) or NS398 (1 μg/5 μl) for 48 h. (**E, F**) In separate experiments, n2a cells were treated with Fe (10 μM) in the absence or presence of M-30 (10 μM) or NS398 (10 μM) for 48 h. The mRNA and protein levels of APH-1α/1β and PS1 were determined by qRT-PCR and western blots, respectively. GAPDH and β-actin served as internal controls. The data represent the means±S.E. of all the experiments. *p<0.05; **p<0.01 compared with C57BL/6 or n2a controls. # *p<0.05* with respect to APP/PS1 or Fe-treated mice alone.

Given the potential roles of Fe and COX-2 in upregulating the production of Aβ, we were prompted to reveal their internal relationship in regulating the expression of APH-1α/1β and PS1. As a first step, we injected an Fe chelator, M-30 (i.c.v, 1 μg/5 μl), into the ventricles of 3-month-old APP/PS1 Tg mice. The results demonstrated that M-30 treatment clearly decreased the expression of APH-1α/1β and PS1 in 3-month-old APP/PS1 Tg mice (Fig. 3A). In this regard, we further determined if COX-2 located downstream of Fe mediates the stimulation of APH-1α/1β and PS1 in APP/PS1 Tg mice. To this end, NS398 was used as a COX-2 specific inhibitor to treat APP/PS1 Tg mice. As expected, NS398 injection (i.c.v, 1 μg/5 μl) significantly inhibited the expression of APH-1α/1β and PS1 in 3-month-old APP/PS1 Tg mice (Fig. 3B). To further verify the key roles of Fe in the above process, we injected (i.c.v) WT mice with Fe (i.c.v, 1 μg/5 μl) in the absence or presence of M-30 (i.c.v, 1 μg/5 μl) for 48 h. The results demonstrated that Fe induced the expression of APH-1α/1β and PS1, which was blocked by the addition of M-30 in 3-month-old WT mice (Fig. 3C). In addition, NS398 (i.c.v, 1 μg/5 μl) also blocked the effects of Fe (i.c.v, 1 μg/5 μl) on the stimulation of the expression of APH-1α/1β and PS1 in 3-month-old WT mice (Fig. 3D). *In vitro* similar experiments were performed to treat n2a cells with Fe (10 μM) in the absence or presence of M-30 (10 μM) or NS398 (10 μM) for 48 h. Our data revealed that Fe located upstream of COX-2 induced the expression of APH-1α/1β and PS1 in n2a cells (Figs. 3E, F). Of note, these observations emphasized the potential role of COX-2 in upregulating the expression of APH-1α/1β and PS1 during the course of AD development and progression.

### The pivotal role of COX-2 in inducing the expression of APH-1α/1β and PS1

**Figure 4:**
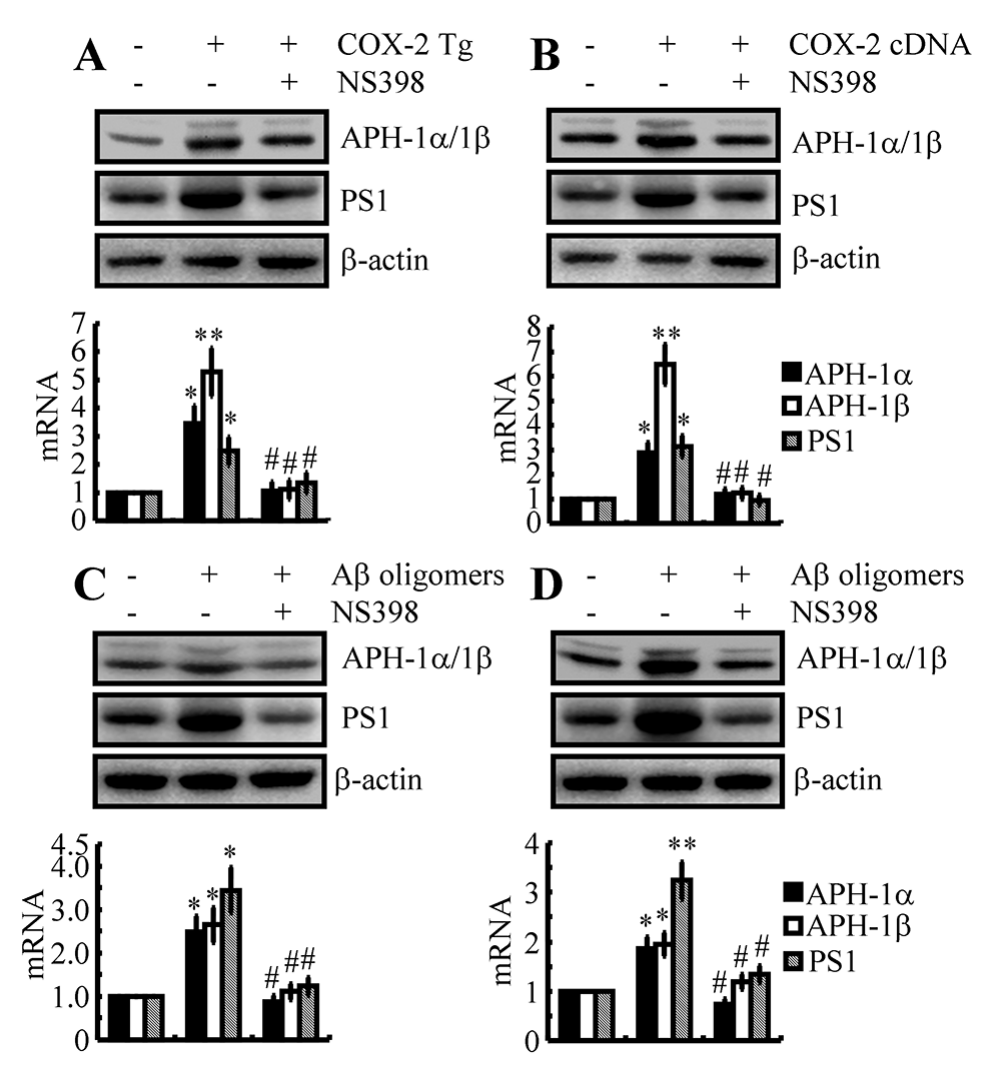
**The key role of COX-2 in regulating the expression of APH-1α/1β and PS1 during the course of AD progression.** (**A**) Three-month-old COX-2 Tg mice were injected (i.c.v) with NS398 (1 μg/5 μl) for 48 h (n=12). (**B**) In select experiments, n2a cells were transfected with COX-2 cDNA constructs in the absence or presence of NS398 treatment (10 μM). (**C**) In separate experiments, C57BL/6 mice were injected (i.c.v) with Aβ (500 ng/5 μl) in the absence or presence of NS398 (1 μg/5 μl) for 48 h. (**D**) In distinct experiments, n2a cells were treated with Aβ oligomers (1 μM) in the absence or presence of NS398 (10 μM) for 48 h. The mRNA and protein levels of APH-1α/1β and PS1 were determined by qRT-PCR and western blots, respectively. GAPDH and β-actin served as internal controls. The data represent the means±S.E. of all the experiments. *p<0.05; **p<0.01 compared with C57BL/6, vector-transfected or vehicle-treated n2a controls. # *p<0.05* with respect to COX-2 Tg mice or Aβ-treated mice alone.

In light of the potential role of COX-2 in mediating the effects of Fe on the induction of APH-1α/1β and PS1 expression, we were prompted to confirm the pivotal role of COX-2 in regulating the expression of APH-1α/1β and PS1. To this end, we injected (i.c.v) COX-2 Tg mice with NS398 (1 μg/5 μl) for 48 h. The results demonstrated that the levels of APH-1α/1β and PS1 were markedly elevated, an effect that was blocked by the injection (i.c.v) of NS398 (1 μg/5 μl) in COX-2 Tg mice (Fig. 4A). To further validate these observations *in vivo*, experiments were carried out *in vitro*. Our data showed that COX-2 overexpression clearly upregulated the expression of APH-1α/1β and PS1 in n2a cells (Fig. 4B). In addition, NS398 (10 μM) treatment clearly suppressed the upregulated APH-1α/1β and PS1 in COX-2-overexpressed n2a cells (Fig. 4B).

Although COX-2 was confirmed to play a critical role in upregulating the expression of APH-1α/1β and PS1, the reasons for COX-2 upregulation are easily questionable in the early stage of AD. As a major pathological characteristic, Aβ is suspected to induce the expression of COX-2 *in vitro* and *in vivo* during the early stage of AD [34]. In reference to this hypothesis, we injected (i.c.v) Aβ oligomers (500 ng/5 μl) into the ventricles of WT mice in the absence or presence of NS398 (i.c.v, 1 μg/5 μl) for 48 h. As expected, the expression of COX-2 was elevated by Aβ oligomers injection (i.c.v, 500 ng/5 μl), an effect that was blocked by the addition of NS398 (i.c.v, 1 μg/5 μl) in WT mice (Fig. 4C). More specifically, these *in vivo* observations were further validated in neurons, and similar results were obtained in n2a cells (Fig. 4D). Taken together, these observations clearly demonstrated that the expression levels of APH-1α/1β and PS1 were elevated as through COX-2 was activated during the course of AD development and progression.

### Involvement of the Tf-TfR system in mediating the effects of Fe on the regulation of the metabolic activity of COX-2 in neurons

**Figure 5:**
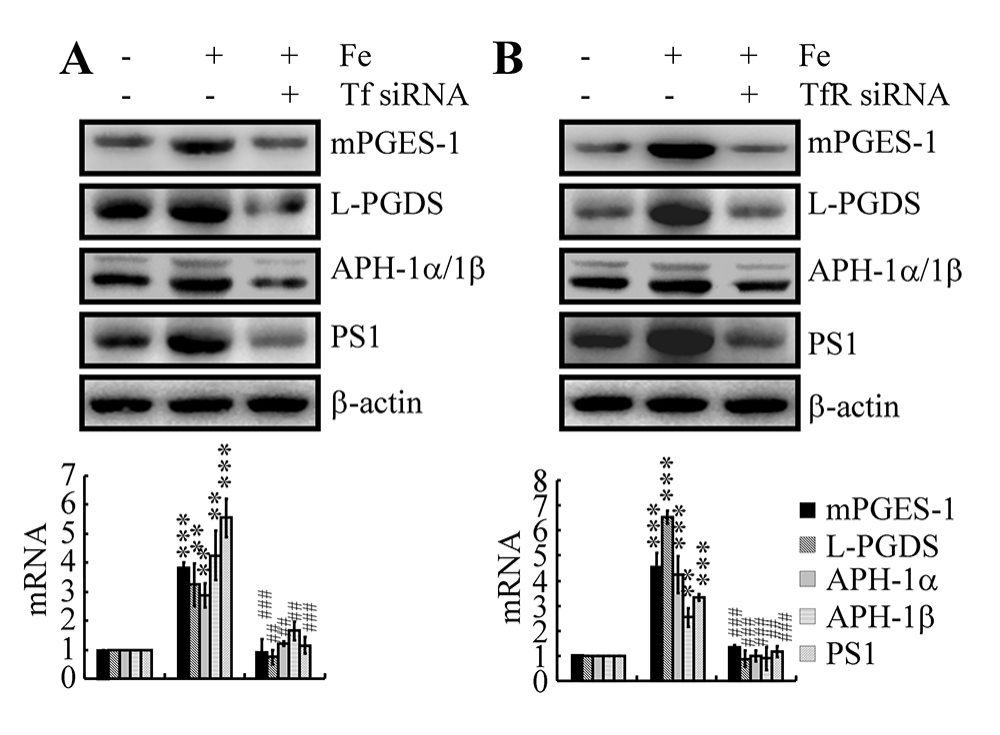
**Tf-TfR mediated the effects of Fe on the stimulation of the expression and metabolic activity of mPGES-1 and L-PGDS, which result in the synthesis of APH-1α/1β and PS1 in neurons.** n2a cells were treated with Fe (10 μM) in the absence or presence of transfection with Tf or TfR siRNA. The mRNA and protein levels of mPGES-1, L-PGDS, APH-1α/1β and PS1 were determined by qRT-PCR and western blots, respectively. GAPDH and β-actin served as internal controls. The data represent the means±S.E. of all the experiments. **p<0.01; ***p<0.001 compared with vehicle-treated controls. ## *p<0.01*; *###p<0.001* with respect to Fe treatment alone.

Although the above data revealed the ability of Fe to upregulate the expression of APH-1α/1β and PS1 by activating COX-2, the mechanism underlying Fe accumulation in neurons and the subsequent actions in the metabolic activity of COX-2 have yet to be defined. To answer these questions, Tf or TfR siRNAs were used to transfect n2a cells. The results demonstrated that knockdown of Tf or TfR expression decreased the stimulatory effects of Fe (10 μM) on the expression of mPGES-1 and L-PGDS in n2a cells (Fig. 5A, B). Although we still could not determine the mechanism of L-PGDS and PGD_2_ upregulation in the late stage of AD, Tf or TfR siRNA transfection clearly decreased the expression of APH-1α/1β and PS1 in Fe-treated n2a cells (Fig. 5A, B). These observations suggested that PGE_2_ and PGD_2_ might be involved in mediating the effects of Fe and the Tf-TfR system on regulating the expression of APH-1α/1β and PS1 in neurons of APP/PS1 Tg mice.

### PGE_2_ and PGD_2_ antagonistically regulate the expression of APH-1α/1β and PS1 in Fe-treated n2a cells

**Figure 6:**
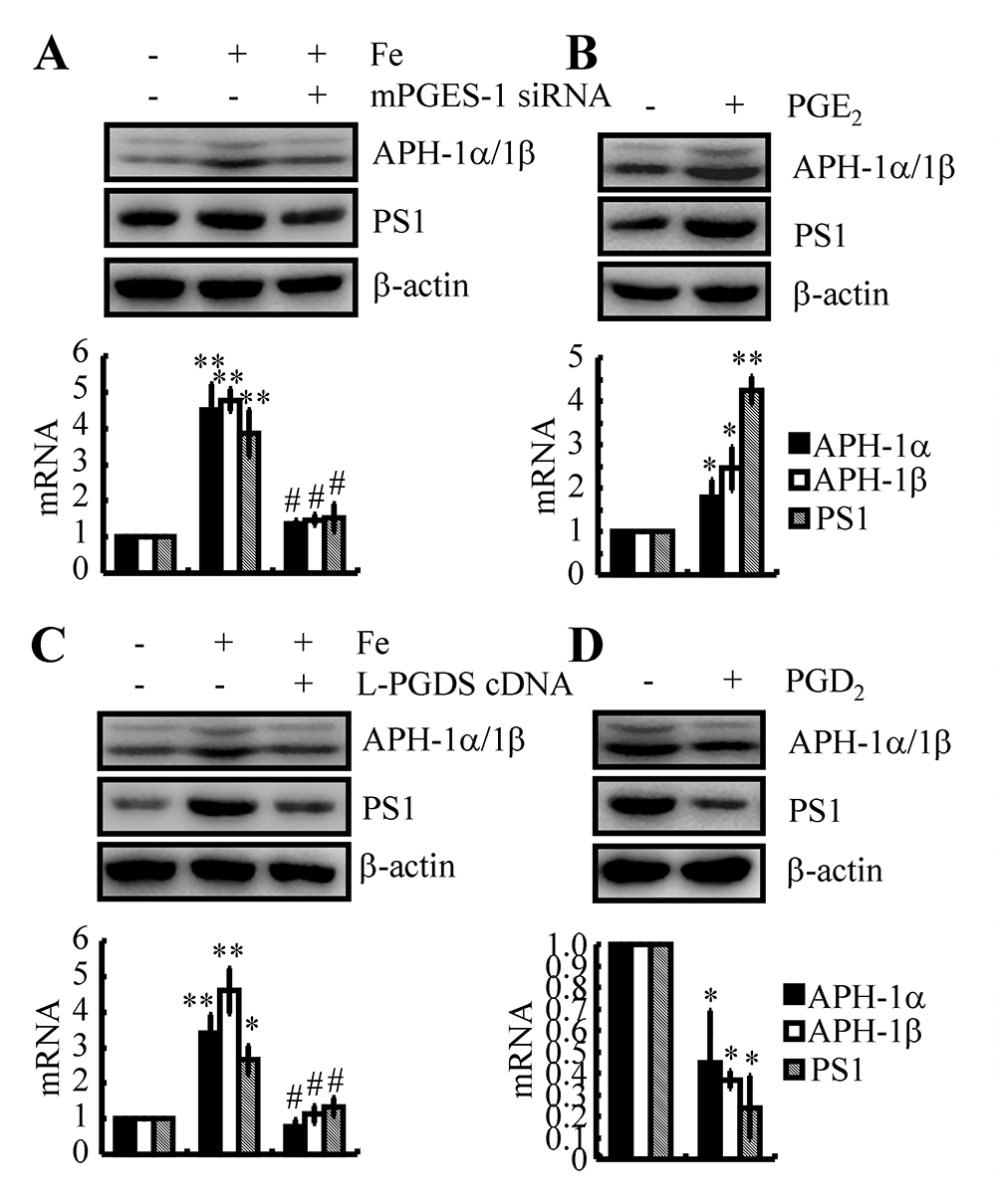
**PGE_2_ and PGD_2_ antagonistically regulated the expression of APH-1α/1β and PS1 in n2a cells.** (**A, C**) n2a cells were treated with Fe (10 μM) in the absence or presence of siRNA-targeted mPGES-1 or L-PGDS cDNA. (**B, D**) n2a cells were treated with PGE_2_ (10 μM) or PGD_2_ (1 μM) for 48 h. APH-1α/1β and PS1 were determined by qRT-PCR and western blots, respectively. GAPDH and β-actin served as internal controls. *p<0.05; **p<0.01 compared with vehicle-treated controls. # *p<0.05* with respect to Fe-treatment alone.

As COX-2 and PGE_2_ are the potential downstream targets of Fe and the Tf-TfR system in the regulation of APH-1α/1β and PS1 expression, we further transfected n2a cells with mPGES-1 siRNA in the presence of Fe. The results demonstrated that mPGES-1 knockdown attenuated the stimulatory effects of Fe (10 μM) on the expression of APH-1α/1β and PS1 in WT mice (Fig. 6A). More specifically, we treated n2a cells with PGE_2_ (10 μM) for 48 h, and the results showed that PGE_2_ upregulated the expression of APH-1α/1β and PS1 in WT mice and n2a cells (Figs. 6B). These observations revealed the essential role of PGE_2_ in upregulating the expression of APH-1α/1β and PS1 in neurons.

Although we still could not determine the mechanism of L-PGDS and PGD_2_ upregulation in the late stage of AD, we could not negate the potential role of PGD_2_ in regulating the expression of APH-1α/1β and PS1 in neurons. To this end, we transfected n2a cells with L-PGDS cDNA plasmids in the absence or presence of Fe (10 μM). The results showed that overexpression of L-PGDS decreased the expression of APH-1α/1β and PS1 in Fe-treated n2a cells (Fig. 6C). Moreover, we treated n2a cells with PGD_2_ (1 μM) for 48 h. The results demonstrated that PGD_2_ treatment for 48 h significantly decreased the expression of APH-1α/1β and PS1 in n2a cells (Fig. 6D). Therefore, PGE_2_ and PGD_2_ showed opposite effects on the regulation of APH-1α/1β and PS1 expression in neurons.

### EP2 and DP1 are critical for mediating the effects of PGE_2_ and PGD_2_ on the regulation of APH-1α/1β and PS1 expression in neurons

**Figure 7:**
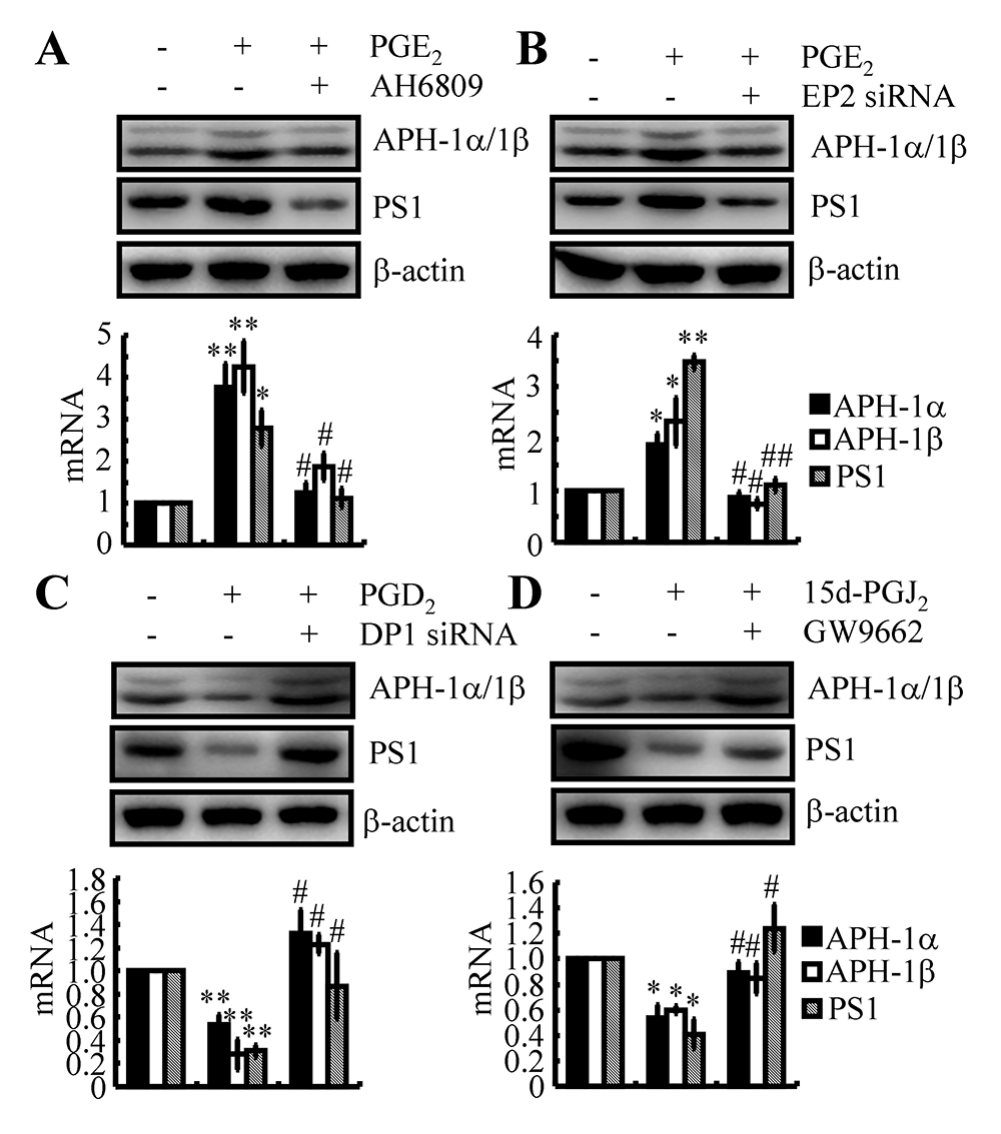
**EP2, DP1 and PPARγ are critical for mediating the effects of PGE_2_, PGD_2_ and 15d-PGJ_2_ on the regulation of APH-1α/1β and PS1 expression in n2a cells.** (**A**) n2a cells were treated with PGE_2_ (10 μM) in the absence or presence of AH6809 (3 μM) for 48 h. (**B**) In select experiments, n2a cells were treated with PGE_2_ (10 μM) in the absence or presence of siRNA specific for EP2. (**C**) In select experiments, n2a cells were treated with PGD_2_ (1 μM) in the absence or presence of siRNA specific for DP1. (**D**) In distinct experiments, n2a cells were treated with 15d-PGJ_2_ (500 nM) in the absence or presence of the PPARγ antagonist GW9662 (1 μM) for 48 h. The levels of APH-1α/1β and PS1 were determined by qRT-PCR and western blots, respectively. GAPDH and β-actin served as internal controls. *p<0.05; **p<0.01 compared with vehicle-treated controls. # *p<0.05*; ## *p<0.01* with respect to PGE_2_-, PGD_2_- or 15d-PGJ_2_-treatment alone.

Since PGE_2_ and PGD_2_ antagonistically regulate the expression of APH-1α/1β and PS1, the receptors involved in mediating the effects of PGE_2_ and PGD_2_ on the expression of APH-1α/1β and PS1 were further elucidated in WT mice and n2a cells. Among EPs, EP2 and EP4 were reported to be involved in increasing the expression of APP and then leading to the accumulation of Aβ by activating γ-secretases. EP2 receptor is enriched in hippocampus and cerebral cortex, EP4 is expressed in thalamic and hypothalamic structures [35]. For the reason that the tissues are collected from cerebral cortex and hippocampus, we thereby select EP4 for the following experiments. Accordingly, we treated n2a cells with PGE_2_ (10 μM) in the absence or presence of AH6809 (3 μM) for 48 h. The results demonstrated that AH6809 treatment clearly blocked the stimulatory effects of PGE_2_ on activating APH-1α/1β and PS1 in n2a cells (Fig. 7A). As nonspecific inhibitor, AH6809 is an EP and DP receptor antagonist with nearly equal affinity for the cloned human EP1, EP2, EP3 and DP1 receptors [36]. In the mouse, AH6809 has the highest affinity for the EP2 receptor but also acts as a weak ligand for the murine DP1 receptor [37]. These observations indicated the potential contributions of EP2 and DP1 to the expression of APH-1α/1β and PS1 in n2a cells. To exclude the nonspecificity of AH6809, we performed a knockdown of the expression of EP2 or DP1 in PGE_2_- or PGD_2_-treated n2a cells. The data revealed that EP2 knockdown blocked the effects of PGE_2_ (10 μM) on stimulating the expression of APH-1α/1β and PS1 in n2a cells (Fig. 7B). In contrast, DP1 knockdown alleviated the suppressive effects of PGD_2_ (1 μM) on the expression of APH-1α/1β and PS1 *in vitro* (Figs. 7C). Of note, CRTH2 was also reported to be the receptor for PGD_2_. Unlike DP1 [38], CRTH2 is not reported to be associated with AD to the best of our knowledge. Based on these findings, we clearly identified EP2 and DP1 as the receptors for PGE_2_ and PGD_2_ in the regulation of APH-1α/1β and PS1 expression in neurons.

### 15d-PGJ_2_ suppressed the expression of APH-1α/1β and PS1 via PPARγ

As the dehydrated product of PGD_2_, 15d-PGJ_2_ is also suspected to be involved in regulating the expression of APH-1α/1β and PS1 in neurons. Regarding this hypothesis, 15d-PGJ_2_ (500 nM) was incubated with n2a cells in the absence or presence of the PPARγ antagonist GW9662 (1 μM). Similar to PGD_2_, 15d-PGJ_2_ treatment inhibited the expression of APH-1α/1β and PS1 in n2a cells (Fig. 7D). As 15d-PGJ_2_ is the natural ligand of PPARγ [39], the addition of the PPARγ antagonist GW9662 alleviated the suppressive effects of 15d-PGJ_2_ on the expression of APH-1α/1β and PS1 in n2a cells (Fig. 7D). These results demonstrated that 15d-PGJ_2_ inhibited the expression of APH-1α/1β and PS1 via a PPARγ-dependent mechanism.

### COX-2 mediated the effects of Fe on the acceleration of the cognitive decline in AD

**Figure 8:**
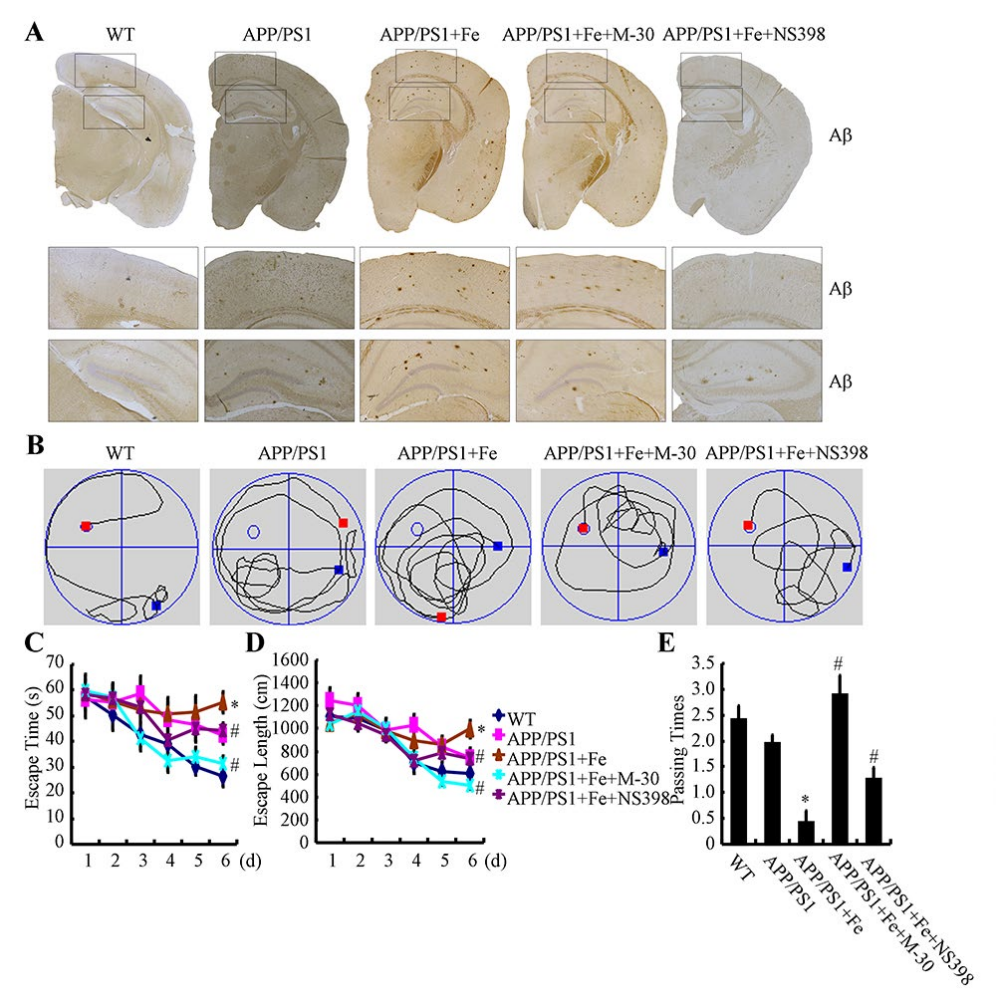
**Fe accelerated cognitive decline in APP/PS1 Tg mice by enhancing the aggregation and deposition of Aβ.** 3-month-old APP/PS1 Tg mice were treated with Fe (25 mg/ml in water) for 3 months in the absence or presence of M-30 (intranasal administration, 0.5 mg/kg/d) or NS398 (intranasal administration, 1 mg/kg/d) before their learning abilities were evaluated (n=6). (**A**) The immunoreactivity of Aβ was determined by immunohistochemistry with an anti-Aβ antibody. These images are representative of 6 independent mouse experiments, all of which produced similar results. (**B-D**) In the hidden-platform tests, Fe-treated APP/PS1 Tg mice showed the longest latency and escape path lengths, whereas those in the M-30 and NS398 treatment groups showed clear decreases in escape latency and path length. (**E**) In the probe trial, the mice in the Fe-treated APP/PS1 group had the fewest times passing through the platform’s former location, and the M-30- or NS398-treated mice showed partially reversed effects of APP/PS1 damage and improved cognition and memory. *p<0.05 compared with APP/PS1 controls. # *p<0.05* with respect to the Fe-treated APP/PS1 mice alone.

In light of the observation that Fe treatment stimulated the expression of APH-1α/1β and PS1, we next investigated the relationship between brain Fe levels and Aβ deposition in APP/PS1 Tg mice. First, we examined the aggregation of Aβ_1-42_ in APP/PS1 Tg mice. The results demonstrated that Fe (25 mg/ml in water) treatment for 3 months clearly induced the deposition of Aβ_1-42_ in the cerebral cortex and hippocampus of APP/PS1 Tg mice (Fig. 8A). Of note, the oral administration of Fe in water significantly increased the serum Fe levels of APP/PS1 Tg mice [29]. More interestingly, a modest increased in Fe levels were observed in Fe-treated APP/PS1 Tg mice though it does not reach a statistically significant [29]. Furthermore, the aggregation of Aβ_1-42_ was disrupted by treatment with M-30 (0.5 mg/kg/d, intranasal administration) or NS398 (1 mg/kg/d, intranasal administration) for 3 months (Fig. 8A). These observations demonstrated that Fe progressively facilitated Aβ_1-42_ deposition in the brains of APP/PS1 Tg mice by activating COX-2.

Apart from Aβ deposition, we further assessed spatial learning and memory abilities by using the Morris water maze task. The results of the pretraining visible-platform tests of the APP/PS1 and Fe-treated APP/PS1 groups did not differ from those of the WT group (data not shown), suggesting that neither APP/PS1 overexpression nor Fe administration had a significant influence on the motility or vision of the C57BL/6 mice. Fe treatment for 3 months prolonged both escape time and length of 6-month-old APP/PS1 Tg mice (Figs. 8B-E). When we performed a probe test 24 h after the last training trial, the Fe-treated APP/PS1 mice showed no preference toward the target quadrant, indicating significant memory impairment, whereas the M-30 or NS398-treated APP/PS1 mice performed as well as the WT C57BL/6 mice (Figs. 8B-E). Taken together, it is clear that Fe accelerates cognitive decline by activating COX-2 via a PGE_2_- and PGD_2_-dependent mechanism.

## DISCUSSION

AD was recently shown to be modulated by dyshomeostasis of Fe in the brain [1,2,40]. However, the underlying mechanism linking Fe and AD remains thoroughly unknown. Therefore, the current investigation was conducted to decipher the stimulatory effects of Fe on AD development. We found that Fe induced the production of Aβ by elevating the expression of APH-1α/1β and PS1 via a COX-2-dependent mechanism. Specifically, Fe stimulated the expression and metabolic activity of COX-2 in a Tf- and TfR-dependent manner, which in turn resulted in abnormal synthesis of PGE_2_ and PGD_2_ via activation mPGES-1 or L-PGDS, respectively. In addition, PGE_2_ and PGD_2_ antagonistically regulated the expression of APH-1α/1β and PS1 via their receptors, including EP2 and DP1, in neurons. Finally, APH-1α/1β and PS1 were found to be responsible for Aβ deposition in neurons during the course of AD development and progression.

Fe has been found to be upregulated during the progression of AD [41]. In addition, an increased understanding of how iron dyshomeostasis is regulated at the whole-body and cellular levels is followed by the identification of a number of iron-related proteins [42–45]. For example, amyloid precursor protein (APP) ferroxidase activity couples with surface ferroportin to export iron, but its activity is inhibited in Alzheimer's disease, thereby causing neuronal iron accumulation [46]. In addition, ferroptosis is recently defined to be critical for cell death in neurological disorders [47]. In particular, the Tf and TfR system seem to play a major role in the homeostasis of Fe [48,49]. Concerning the Tf gene, a number of studies have shown various relationships of Tf changes in AD, but the data are still controversial and far from univocal interpretation. For example, it was initially reported that the levels of Tf in the serum of 41 AD patients tended to be lower than those of 19 age-matched subjects [50]. In contrast to this observation, a recent meta-analysis identified 5 separate studies with a total of 153 AD patients and 545 controls, of which 2 reported decreases in plasma iron levels, while the remaining 3 failed to identify significant changes [51]. This report suggested that the subtle changes in a specific Fe-binding protein in the circulatory system might not be enough to affect the pathogenesis of AD. Although Tf has 16 variants, the Tf C1 and C2 variants account for the majority of the population in all races [52]. As the most prevalent variant of Tf, Tf C1 expression has been found to remain constant during the course of AD development and progression. In contrast to Tf C1, a significant increase in Tf C2 was found to occur in AD patients [53]. In agreement with this observation, we also found that Tf C2 expression was elevated in 3-month-old APP/PS1 Tg mice. This observation indicated the potential role of Aβ in upregulating the expression of Tf in neurons. Regarding this hypothesis, Aβ has the ability to stimulate the expression of Tf via a CP2-dependent mechanism [54]. In more detail, Aβ_1-42_ and Aβ_25-35_ increased the binding activity of CP2 to the promoter of Tf, which results in mRNA and protein expression of Tf [55]. These observations were coincided with the phenomenon showing that Tf was significantly increased in AD frontal cortex, compared with elderly controls [56]. In addition, TF C2 associated with two HFE mutations, namely, C282Y and H63D, has been reported as a potential risk factor for AD [57]. For instance, it has been hypothesized that highly expressed Tf C2 and the mutation of HFE H63D may lead to the onset of AD [58]. Other authors evaluated the possible interaction between Tf C2 and HFE C282Y, which results in Fe transport and the onset of AD [59]. Even in the early stage of AD, the combination of Tf C2 and HFE C282Y has the ability to transport an excess of redox-active Fe^2+^, which induces mild cognitive impairment (MCI) [60]. Along these lines, we further found that Tf C2 is critical for mediating Fe-induced production of Aβ during the course of AD development.

In light of the critical role of Tf C2 in the pathogenesis of AD, we continued to elucidate its receptor (TfR) for Aβ deposition as part of the critical role of TfR in internalizing the Fe-bound transferrin complex [20]. The results demonstrated that TfR knockdown blocked Fe-induced expression of APH-1α/1β and PS1, which resulted in the suppression of Aβ in APP/PS1 Tg mice. In agreement with our data, Huang *et al.* [61] reported that the expression of TfR1 was elevated in APP/PS1 Tg mice, an effect that was critical for the production and deposition of Aβ. In addition, treatment of APP/PS1 Tg mice with an antibody specific for TfR1 decreased the aggregation of Aβ [62,63]. However, the expression of TfR1 is not always upregulated during the course of AD development and progression. There is a report suggesting that TfR1 are upregulated at the early stage of AD, such as 3-month-old APP/PS1 Tg mice [64], whereas the expression of TfR1 begins to decrease at the age of 6-month-old [64]. These prior works are consistent with our observations suggesting that Tf and TfR are elevated in 3-month-old APP/PS1 Tg mice. To the reason, it should be caused by the production of Aβ since the only difference between WT and APP/PS1 Tg mic is the excessive loading of Aβ in the brains of APP/PS1 Tg mice. In addition, the non-aggregated form of Aβ should play key roles in upregulating the expression of TfR since the expression of TfR in APP/PS1 Tg mice is downregulated significantly after postnatal 6 months compared with WT mice [64]. Along these lines, it is clear that Tf C2 and the TfR1 system play key roles in mediating Fe-induced the production of Aβ at the early stage of AD.

Although Tf C1 and TfR1 are involved in regulating Aβ deposition, the key molecule for the pathogenesis of AD is the accumulation of Fe during the course of AD development and progression. Fortunately, we found that Fe has the ability to induce the expression of APH-1α/1β and PS1 in pathological neurons. In concert with our observation, intranasal administration of DFO has been reported to block the effects of Fe on inducing amyloidogenic APP processing [29] and the production of Aβ in APP/PS1 mice [65]. In detail, Fe treatment does not notably alter the production of sAPPα, but the level of sAPPβ in Fe-treated group increased 157.99±16.63% (*p<0.01*). Furthermore, the levels of C99 are significantly elevated in the brains of Fe-treated mice, and the levels of C83 fragments were not changed so much in Fe-treated mice [29]. More closely, Matsuzaki *et al.* [66] reported that Fe accelerated the production of the aberrant splicing isoform of PS2. Reciprocally, Greenough *et al.* [67] summarized the role of PS in the trafficking and degradation of transporters, which result in the regulation of homeostasis of metal ions, such as Fe. Along these lines, our data extended prior works to reveal the activity of Fe in regulating the activity of γ-secretases.

As Fe potentially contributes to the expression and metabolic activity of COX-2 in rats with diabetic nephropathy [22], we continued to elucidate the role of Fe in regulating the production of Aβ via a COX-2-activating mechanisms. As expected, COX-2 was involved in modulating the expression of APH-1α/1β and PS1 during the course of AD development. In agreement with our observation, PGE_2_ was found to play a critical role in the regulation of BACE-1 [68–70] and PS1 [71] expression, which results in Aβ deposition in different experimental systems. In contrast, PGE_2_ exerts positive effects on the regulation of ADAM-10 expression in mouse gastric tumors [72], but to the best of our knowledge, the effects of PGE_2_ on ADAM-10 expression have not been documented in neuronal systems. Based on these observations, we further found that PGD_2_ and 15d-PGJ_2_ suppressed the expression of APH-1α/1β and PS1 in neurons of APP/PS1 Tg mice. Although we could not find any direct evidence of a relationship between PGs and APH-1α/-1β, particularly over the course of AD development, APH-1α/-1β has been regarded as a biomarker of AD. For instance, APH-1 was reported to combine with PEN-2, nicastrin, and PS to generate an active form of the γ-secretase complex that cleaves β-APP and deposits Aβ [73]. More specifically, we found that APH-1α/1β and PS1 are binding partners for producing deposition of Aβ in APP/PS1 Tg mice [33]. Therefore, APH-1α/1β and PS1 are critical for mediating the antagonistic effects of PGE_2_ and PGD_2_ on the regulation of Aβ production during the course of AD development and progression.

In light of the essential roles of APH-1 and PS1 in Aβ production, our data further revealed that 15d-PGJ_2_ treatment inhibited the expression of APH-1 and PS1 in a PPARγ-dependent manner. Consistent with our observations, Kummer *et al.* [74] recently reported that oral treatment of APP/PS1 mice with the novel PPARα/β/γ agonist GFT1803 decreases Aβ levels and the area affected by APs. More specifically, PPARγ agonist treatment was shown to improve cognitive decline in Tg2576 APP mice by normalizing dentate granule cell presynaptic function [75]. Prakash and Kumar [76] further found that a PPARγ agonist (pioglitazone), but not the antagonist bisphenol A diglycidyl ether (BADGE), protects Wistar rats from the cognitive impairments caused by Aβ injection. This observation was further validated through the use of another PPARγ agonist, rosiglitazone, and the PPARγ antagonist GW9662 in cultured neurons [77]. On the basis of these prior works, we further filled these gaps regarding PPARγ and Aβ by identifying the molecules APH-1α/1β and PS1. Taken together, our results elucidated the mechanisms by which Fe stimulates the production and deposition of Aβ by inducing the expression of APH-1α/-1β and PS1 aggregation via a Tf- and TfR-dependent COX-2 activation mechanism.

## MATERIALS AND METHODS

### Reagents

PGE_2_, PGD_2_, Fe_2_(SO_4_)_3_ and the inhibitors including AH6809 and GW9662 were obtained from Sigma-Aldrich Corp (St. Louis, MO, USA). M-30 was also obtained from Sigma-Aldrich Corp (St. Louis, MO, USA). 15d-PGJ_2_ were obtained from Enzo Life International Inc. (Plymouth Meeting, PA). NS398 was purchased from Tangchao Chemical Co., Ltd. (Xi’an, China). Aβ was synthesized by Qiangyao Biotechnology (Shanghai, China). mPGES-1, EP2, DP1, Tf, TfR or scramble siRNA and antibody specific for Tf or TfR were obtained from Santa Cruz Biotechnology (Santa Cruz, CA, USA). COX-2 or L-PGDS cDNA plasmids were obtained from Origene Technologies (Rockville, MD, USA), and subcloned to the pCMV6-XL vector. Antibodies against β-actin, PS1 and Aβ (Stock No. 15126) were purchased from Cell Signaling Technology, Inc. (Danvers, MA, USA). mPGES-1 and L-PGDS antibody was obtained from Sigma-Aldrich Corp (St. Louis, MO, USA). Antibody specific for APH-1α/1β was purchased from Merck Millipore (Bedford, MA, USA). All reagents for the qRT-PCR and SDS-PAGE experiments were purchased from Bio-Rad Laboratories. All other reagents were from Invitrogen (Carlsbad, CA, USA) unless otherwise specified.

### Cell culture

Mouse neuroblastoma 2a (n2a) cells were grown (37 °C and 5% CO_2_) on 6-cm tissue culture dishes (10^6^ cells per dish) in appropriate medium. In a separate set of experiments, the cells were grown in serum-free medium for an additional 12 h before incubation with inhibitors in the absence or presence of PGE_2_ or PGD_2_, as previously described [70].

### Transgenic mice and treatments

The wild type (WT) or APP/PS1 (B6C3-Tg (APPswe, PSEN1dE9) 85Dbo/J (Stock Number: 004462)) or COX-2 (C57BL/6-Tg(Thy1-PTGS2)303Kand/J (Stock Number: 010703)) Tg mice were obtained from The Jackson laboratory (Bar Harbor, ME, USA). Genotyping was performed at 3-4 weeks after birth. The mice were housed in a controlled environment under a standard room temperature, relative humidity and 12-h light/dark cycle with free access to food and water. Mice at 3 months of age were injected (i.c.v) with M-30 or NS398 for 48 h before collecting the brains of mice. The general health and body weights of animals were monitored every day. The brains of animals from the different groups were collected under anesthesia and perfusion as previously described [32,33,78,79].

### Real-Time PCR

qRT-PCR assays were performed with the MiniOpticon Real-Time PCR detection system (Bio-Rad) using total RNA and the GoTaq one-step Real-Time PCR kit with SYBR green (Promega) and the appropriate primers as previously described [32,80,81]. The GenBank accession number and forward and reverse primers for mouse APH-1α, APH-1β, PS1 and GAPDH are pro-vided in our previous publications [32,80,81]: mouse mPGES-1 (NM_022415) F-gcacactgctggtcatcaag, R-ggt tgggtcccaggaatgag; L-PGDS (NM_008963) F-cacagtgc agcccaactttc, R-gggctgctgtaggtgtagtg; Tf (NM_133977) F-tttttcagtcaaggctgcgc, R-tcggcagggttctttccttc; TfR (NM_011638) F-cgaagtccagtgtgggaaca, R-aaggggctggc agaaacttt. The gene expression values were normalized to those of GAPDH. The ratio was claculated by the following equation:

Ratio=$\frac{2^{∆Ct\left({Gene}_{wt}-{Gene}_{\frac{APP}{PS1}}\;\;\right)\;\;\;\;\;}}{2^{∆Ct\left({GAPDDH}_{wt}-{GAPDH}_{\frac{APP}{PS1}}\right)\;\;\;\;\;\;\;.\;}}$

### Western blots

Western blots were performed as previously described [33,82–86]. In brief, tissues or cells were lysed in radioimmune precipitation assay buffer (25 mM Tris-HCl [pH 7.6], 150 mM NaCl, 1% NP-40, 1% sodium deoxycholate, and 0.1% SDS) containing protease inhibitor cocktail (Pierce Chemical Company). The protein content of the cell lysates was determined using a bicinchoninic acid (BCA) protein assay reagent (Pierce Chemical Company). The total cell lysates (4 μg) were subjected to SDS-PAGE, transferred to a membrane, and probed with a panel of specific antibodies. Each membrane was only probed with one antibody. β-actin was used as a loading control. All western hybridizations were performed at least in triplicate using a different cell preparation each time.

### Aβ_1-42_ preparation

The methods for preparing Aβ oligomers had been described previously [87]. In brief, freeze-drying Aβ_1-42_ protein (Stock Number: A9810, Sigma, St. Louis, MO, USA) was initially monomerized by dissolving it to a final concentration of 1 μg/μl in 100% hexafluoroisopropanal (HFIP) and the solution was aliquoted in sterile eppendorf tubes. HFIP was then evaporated under vacuum and the peptide was stored at -20 °C before reconstituent. For preparing Aβ_1-42_ oligomers, the peptide was initially resuspended in dimethylsulfoxide (DMSO) to 20 μg/μl with water bath ultrasonication for 10 min and the solution was then diluted to a final concentration of 0.2 μg/μl in phenol red-free F-12 media and incubated at 4 °C for 24 h before use.

### Intracerebroventricular injection

NS398, PGE_2_, PGD_2_ or vehicle (PBS) were intracerebroventricularly injected (i.c.v) to WT, APP/PS1 or COX-2 Tg mice as previously described [32,33,78,79]. Briefly, stereotaxic injections were placed at the following coordinates from the bregma: mediolateral, -1.0 mm; anteroposterior, -0.22 mm; and dorsoventral, -2.8 mm. Following injection, each mouse recovered spontaneously on a heated pad. The reliability of the injection sites were validated by injecting trypan blue dye (Invitrogen) in separate cohorts of mice and observing staining in the cerebral ventricles. 48 h after injection, mice were harvested after anesthesia and perfusion [32,33,78,79].

### Immunohistochemistry

Mouse brains were collected from WT or APP/PS1 Tg mice and immobilized with 4% paraformaldehyde. Serial sections (10 μM thick) were cut using a cryostat (Leica, CM1850, Germany). Slides were first rehydrated in a graded series of ethanol and submerged in 3% hydrogen peroxide to eliminate endogenous peroxidase activity. Levels of Tf, TfR and Aβ were determined using an immunohistochemical staining kit, following the manufacturer’s instructions (Invitrogen) as previously described [32,33,78,79].

### Morris water maze test

The mice were trained and tested in a Morris water maze according to the previous description [33]. In brief, the mice were pretrained in a circular water maze with a visible platform for 2 d. The platform was then submerged inside the maze, with the deck 0.5 cm below the surface of the water for the following experiments. Milk was added to the water to hide the platform from sight. The mice were placed inside the maze to swim freely until they found the hidden platform. The whole experiment lasted for 7 d. For the first 6 d, the mice were left in the maze with a maximum time of 60 s to find the platform. The learning sessions were repeated for 4 trials each day, with an interval of 1 h between each session. The spatial learning scores (the latency period needed to find and climb onto the hidden platform and the length of the path to the platform) were recorded. On the last day, the platform was removed, and the times that the mice passed through the memorized region were recorded for a period of 2 min (120 s). Finally, the recorded data were analyzed with a statistical program (ZH0065; Zhenghua Bioequipment, Yuanyang City, Henan, China).

### Animal committee

All animals were handled according to the care and use of medical laboratory animals (Ministry of Health, Peoples Republic of China, 1998), and all experimental protocols were approved by the Laboratory Ethics Committees of China Medical University and the College of Life and Health Sciences of Northeastern University.

### Statistical analysis

All data are represented as the mean±S.E. of at least three independent experiments. The statistical significance of the differences between the mean values was determined with Student’s t-tests or 1-way or 2-way analysis of variance (ANOVA), as appropriate [32,33,78,79].
